# Mitochondrial biogenesis dysfunction and metabolic dysfunction from a novel mitochondrial tRNA^Met^ 4467 C>A mutation in a Han Chinese family with maternally inherited hypertension

**DOI:** 10.1038/s41598-017-03303-w

**Published:** 2017-06-08

**Authors:** Yuqi Liu, Yang Li, Chao Zhu, Liuyang Tian, Minxin Guan, Yundai Chen

**Affiliations:** 1 Cardiac department of Chinese PLA General Hospital, Beijing, 100853 China; 2 Institute of Geriatric Cardiology of Chinese PLA General Hospital, Beijing, 100853 China; 3 Cardiac department of People’s Hospital of Tianjing, Tianjing, 300121 China; 4 Institute of Genetics, Zhejiang University and Department of Genetics, Zhejiang University, School of Medicine, Hangzhou, Zhejiang, 310058 China

## Abstract

To investigate the relationship between mitochondrial DNA (mtDNA) and hypertension as well as the mechanism involved in mitochondrial metabolic dysfunction. We identified a novel tRNA^Met^ C4467A mutation in a Han Chinese family with hypertension. The maternal members presented with increased glucose, total cholesterol, low-density lipoprotein, and serum sodium as well as decreased potassium compared with non-maternal members (*P* < 0.05). Segregation analysis showed this mutation was maternally inherited. We analyzed lymphocyte cell lines derived from three maternal and three non-maternal family members. Reactive oxygen species production in the mutant cell lines was 114.5% higher compared with that in controls (*P* < 0.05) while ATP was 26.4% lower. The mitochondrial membrane potential of the mutated cell lines was 26.2% lower than that in controls (*P* < 0.05). Oxygen consumption rates were decreased in the mutant cell lines (*P* < 0.05). The activation of caspase-3/7 was 104.1% higher in the mutant cell lines compared with controls (*P* < 0.05). The expression of voltage-dependent anion channel (VDAC), Bax and apoptosis-inducing factor (AIF) in the mutant cell lines was higher compared with that in controls, with the increased colocalization of VDAC and Bax. Therefore, this mutation contributes to oxidative stress and mitochondrial biogenesis dysfunction, which may be involved in the pathogenesis of hypertension.

## Introduction

Hypertension is a common risk factor for many cardiovascular diseases, including coronary heart disease, heart failure, stroke and renal disease^1^. Essential hypertension is known as a multifactorial disease with both genetic and environmental factors affecting its onset and severity. Estimates of genetic variance range from 20–50%^2–4^, and both maternal and paternal patterns have been reported^5–7^. Significant blood pressure (BP) correlations between excess maternal transmission and mother-and-offspring hypertension have been observed^5, 8^. Indeed, we have previously reported the excess maternal transmission of hypertension in hypertensive families^9–14^ with the identification of several mitochondrial DNA (mtDNA) mutations, including T3308C in *ND1* ^9^, A4435G in transfer tRNA^Met^ ^11^, A4263G in tRNA^Ile^ ^13^, and A4401G in tRNA^Met^ and tRNA^Gln^ ^12^. We showed that these mutations lead to a decrease in tRNAs and several polypeptides encoded by mtDNA^11–13^. However, the relationship between mtDNA mutations and high BP remains unclear.

To investigate the effect of mtDNA mutations on hypertension pathogenesis, we performed a systematic mtDNA mutational screening in a large cohort of hypertensive subjects in the Geriatric Cardiology Clinic at the Chinese PLA General Hospital in China^9–14^. Here, we reported on a Han Chinese family with maternally inherited hypertension and the novel mutation tRNA^Met^ C4467A. We carried out a systematic investigation of changes in oxidative stress and mitochondrial biogenesis that are associated with the tRNA^Met^ C4467A mutation to further explore the possible mechanisms of mtDNA mutation pathogenesis in maternally inherited hypertension.

## Results

### Pedigree analysis

**Figure 1:**
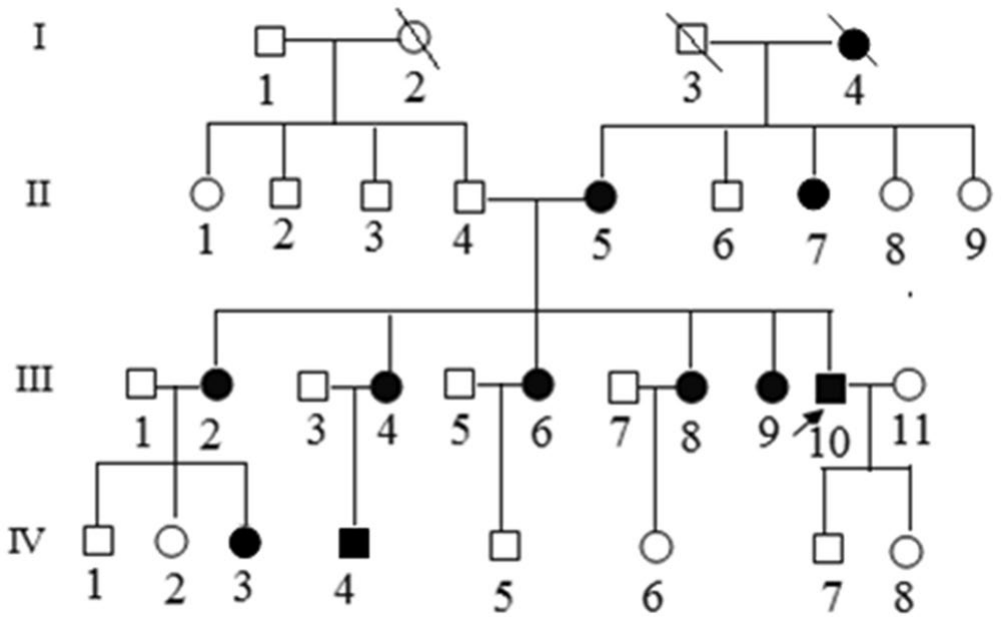
The Han Chinese family with hypertension carrying the mitochondrial tRNA^Met^ 4467 C>A mutation. Arrowhead denotes the proband. Affected individuals are indicated by filled symbols.

The proband (III-10), a 42-year-old male, underwent a health evaluation at the clinic and was found to have borderline hypertension with a BP of 140/90 mmHg. He did not smoke or drink alcohol and did not have a history of coronary artery disease, diabetes, or hyperlipidemia. The pedigree originated from Shan Xi Province, China (Fig. 1). The proband’s mother and five sisters also presented with hypertension. Statistical analysis of the age of hypertension onset of relatives on the maternal lineage showed that the average age of onset for the second generation was 62.0 ± 6.2 years, while the average ages were younger for the third generation (46.3 ± 5.8 years) and fourth generation (23.3 ± 2.9 years) (Table 1). The blood pressure levels in this family are listed in Table 2.

**Table 1:** comparison of age of onset and blood pressure of the individuals on maternal lineage.

Generation	Age of onset (year)	SP (mmHg)	DP (mmHg)
II	62.0 ± 6.2	157.5 ± 15.0	80.0 ± 11.5
III	46.3 ± 5.8	137.2 ± 16.4	90.6 ± 10.1
IV	23.3 ± 2.9	127.1 ± 18.2	87.9 ± 11.5

**Table 2:** Clinical Data for Some Members in a Large Chinese Pedigree.

Subjects	Gender	Age of Test (yrs)	Age of Onset (yrs)	Systolic Pressure (mmHg)	Diastolic Pressure (mmHg)
II-1	F	65	—	135	80
II-2	M	67	—	135	60
II-3	M	69	—	128	60
II-4	M	70	—	125	70
II-5	F	69	57	147	88
II-6	M	70	—	130	60
II-7	F	71	66	168	72
II-8	F	73	—	130	65
II-9	F	74	—	125	70
III-1	M	54	—	125	75
III-2	F	52	52	168	85
III-3	M	53	—	130	75
III-4*	F	51	51	124	85
III-5	M	55	—	124	60
III-6*	F	50	49	125	78
III-7	M	51	—	128	60
III-8	F	48	47	136	105
III-9	F	40	37	130	100
III-10	M	42	42	140	90
III-11	F	40	—	120	70
IV-1	M	21	—	126	60
IV-2	F	23	—	125	68
IV-3*	F	26	25	114	80
IV-4	M	25	21	140	96
IV-5	M	25	—	128	64
IV-6	F	21	—	120	70
IV-7	M	16	—	110	65
IV-8	F	20	—	105	60

^*^These patients accept oral antihypertension treatment. This table shows post-treatment blood pressures.

We performed segregation analysis to differentiate between different patterns of inheritance, including autosomal recessive, autosomal dominant, X-linked, or mitochondrial inheritance^8^, as shown on the pedigree in Fig. 1. Autosomal inheritance would be indicated by an equal number of hypertensive offspring from hypertensive fathers compared to hypertensive mothers. However, 10 out of 17 offspring from hypertensive mothers exhibited hypertension, while both offspring of hypertensive fathers did not exhibit hypertension. Therefore, it is not consistent with autosomal inheritance. X-linked recessive inheritance would be indicated by a higher number of affected males than affected females. However, a difference was not observed between the number of affected females and males in this family, and therefore, an X-linked recessive inheritance pattern was also rejected. X-linked dominant inheritance would be indicated by hypertension in all daughters of hypertensive fathers, which was also excluded in this family. Therefore, the family presented with maternal inheritance, which could be explained by mitochondrial or imprinting inheritance. Imprinting was excluded because the ratio of affected offspring from hypertensive mothers should have been less than or equal to 0.5 l however, the ratio for this family was 0.56 (10 out of 18). Thus, mitochondrial DNA mutation was the most likely explanation of the hypertension etiology in this pedigree.

### Biochemical analysis of maternal and non-maternal individuals showed metabolic dysfunction

Blood specimens of 28 individuals from this pedigree were obtained for biochemical testing. Statistical analysis showed that individuals from the maternal lineage had significant increases in total cholesterol (TC), low-density lipoprotein (LDL), blood glucose and serum sodium as well as a decrease in potassium compared with non-maternal relatives (*P* < 0.05, Table 3 and Supplemental Fig. 1).

**Table 3:** Biochemical test for members in this Chinese Pedigree.

Subjects	Glucose (mmol/L)	TC (mmol/L)	LDL (mmol/L)	Potassium (mmol/L)	Sodium (mmol/L)
II-1	3.22	3.59	2.49	4.23	136.1
II-2	2.54	4.12	3.05	4.34	134.5
II-3	2.05	3.72	2.24	3.87	134.8
II-4	3.64	4.61	3.06	4.56	129.4
II-5	4.77	6.53	5.44	3.51	138.2
II-6	6.20	4.28	3.21	3.52	137.9
II-7	4.45	5.88	4.59	3.91	136.7
II-8	5.44	3.99	3.01	3.49	132.6
II-9	5.28	5.15	4.12	4.20	135.8
III-1	2.41	4.21	3.45	3.69	133.6
III-10	4.05	6.12	5.91	3.68	134.8
III-11	2.62	4.17	3.25	4.91	132.8
III-2	12.62	7.01	5.94	4.50	136.5
III-3	2.59	4.26	3.23	8.44	135.2
III-4	3.20	4.99	3.58	3.56	133.3
III-5	3.97	3.75	2.35	3.97	135.8
III-6	5.06	4.54	3.54	4.21	142.6
III-7	2.98	3.48	2.48	4.12	134.4
III-8	5.00	4.10	3.08	4.50	139.0
III-9	5.65	6.44	5.87	3.66	132.2
IV-1	2.58	3.09	2.00	4.33	133.6
IV-2	2.71	3.10	2.09	3.45	138.1
IV-3	1.98	2.81	2.01	4.33	134.1
IV-4	4.68	3.22	2.11	4.24	137.6
IV-5	3.80	3.87	2.33	3.56	136.5
IV-6	3.22	3.59	2.59	4.23	136.1
IV-7	3.18	3.25	2.01	7.16	135.5
IV-8	2.84	3.02	1.98	5.25	131.5

TC: total cholesterol; LDL: low-density lipoprotein.

### mtDNA sequence analysis and tRNA 2-D structure analysis

**Figure 2:**
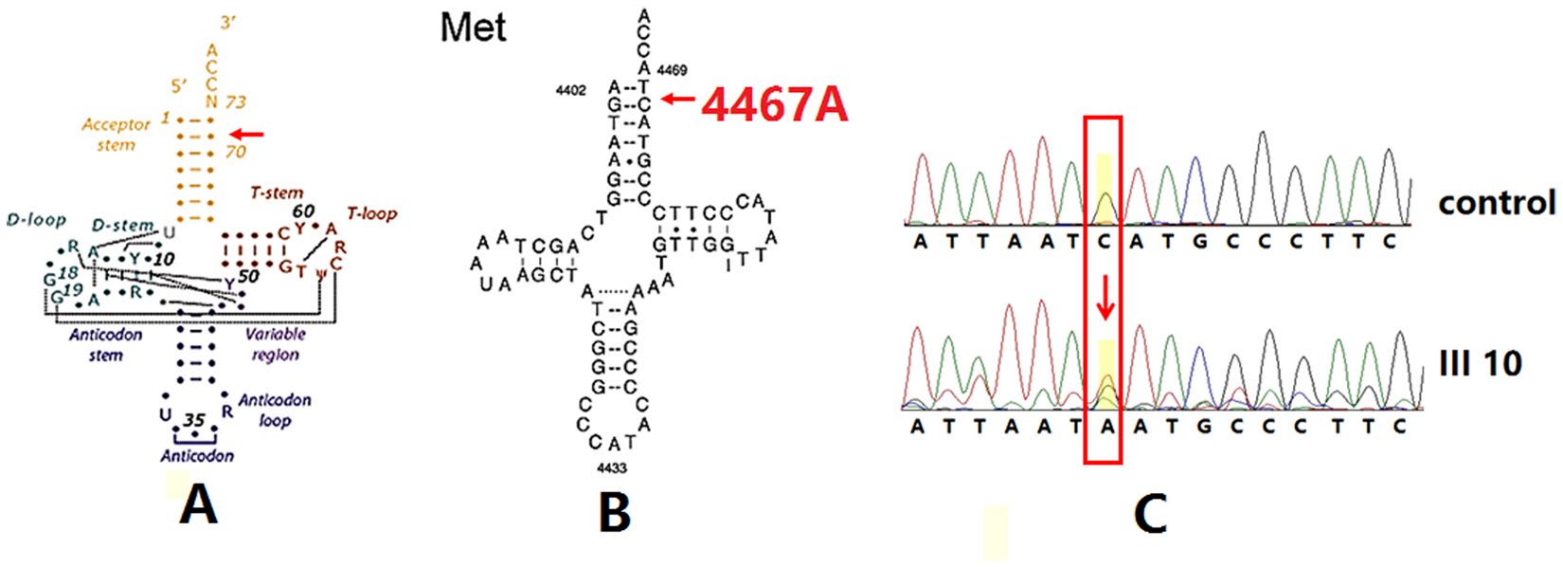
Identification and qualification of the 4467 C>A mutation at the 3′ end of the acceptor arm. (**A**) Secondary structure of tRNA: acceptor stem, D-stem, D-loop, anticodon stem, anticodon loop, T-stem, and T-loop. (**B**) The 4467 C>A mutation is located in the precursors of tRNA^Met^. Arrow indicates the position of the 4467 C>A mutation. (**C**) Partial sequence chromatograms including tRNA^Met^ 4467 genes from hypertensive individual (III-10) and a married-in control (III-11).

Because the transmission pattern of hypertension in this family appeared to be maternal inheritance, mtDNA may be involved in the disease pathogenesis. We therefore analyzed the whole mitochondrial genome of members in this family using PCR amplification and subsequent sequence analysis of the PCR fragments. The tRNA^Met^ C4467A mutation was identified in the proband, as shown in Fig. 2. Sequencing of PCR fragments revealed that the tRNA^Met^ C4467A mutation was absent from 366 Chinese controls. All of the other 16 members of the maternal lineage were shown to carry the tRNA^Met^ C4467A. This mutation is located at the 3′ end of the tRNA^Met^, which is the processing site for the tRNA^Met^ 3′-end precursors of the light strand and is predicted to alter A–U base pairing (A1-U72) at the aminoacyl acceptor stem of tRNA^Met^.

tRNA variants were also compared by phylogenetic analysis of sequences from other organisms, including the Mexican fruit bat *Artibeus jamaicensis*, the wild boar *Sus scrofa*, mice^15^, cows^16^, and *Xenopus laevis* ^17^ (Supplemental Fig. 2). None of the other variants were highly evolutionarily conserved, except for tRNA^Met^ C4467A, suggesting that this site has an important functional role.

### The tRNA^Met^ C4467A mutation contributes to an increase of ROS production

**Figure 3:**
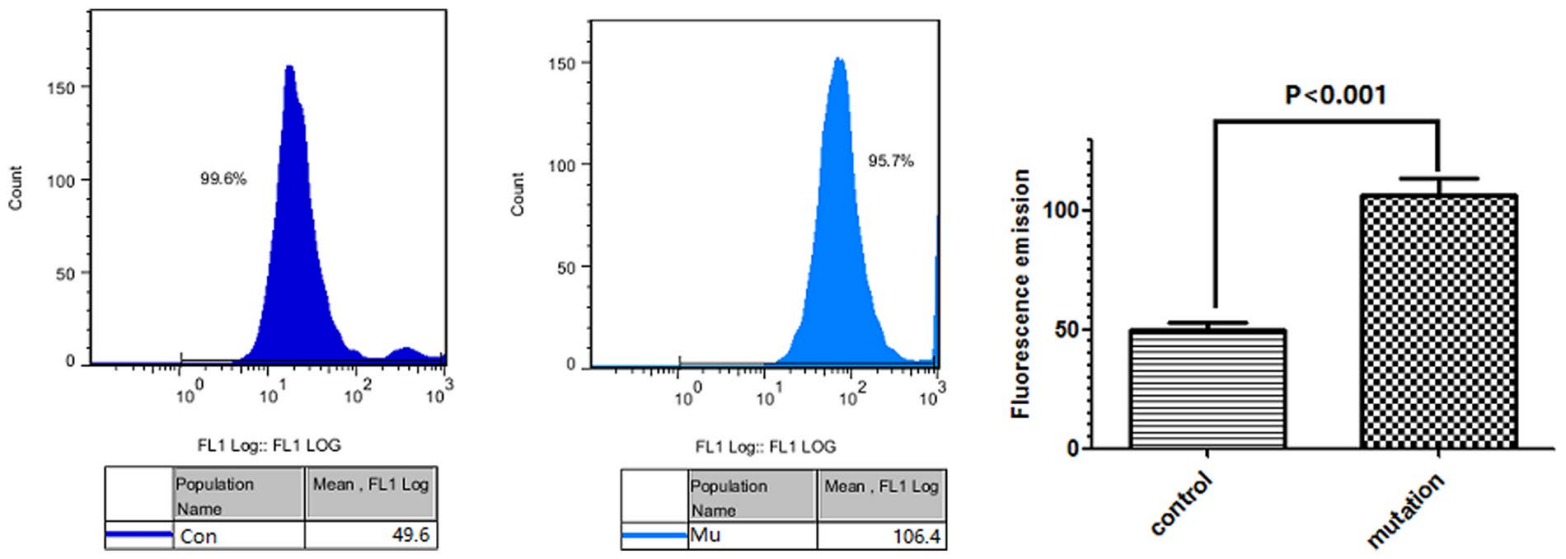
ROS level evaluation in lymphocytes from controls and the tRNA^Met^ 4467 C>A mutation group. Levels of ROS in lymphocytes were evaluated with DCFH-DA (10 μM) fluorescence staining using a flow cytometer. The fluorescence level of the controls was 49.6 and that of the tRNA^Met^ 4467 C>A mutation group was 106.4; the difference was statistically significant (*P* < 0.05).

DCFH-DA probe-labeled cells were used to detect the effect of the mitochondrial tRNA^Met^ C4467A mutation on intracellular ROS production by flow cytometry. Cell lines carrying tRNA^Met^ C4467A showed significantly higher ROS production than control cell lines (Fig. 3, 49.6 ± 3.7 vs. 106.4 ± 6.8, *P* < 0.001).

### The tRNA^Met^ C4467A mutation contributes to a decrease in ATP concentration

We used a bioluminescence-based cell viability assay for ATP measurements. The cell concentration gradient of 1 × 10^3^, 2 × 10^3^, 4 × 10^3^, and 8 × 10^3^ cells presented with a linear relationship with ATP concentrations both for the cells carrying the tRNA^Met^ C4467A mutation and the control cell lines (Supplemental Fig. 3B). ATP production of the tRNA^Met^ C4467A-mutated lymphocyte cell lines was significantly lower than that of control cell lines for groups with the same cell number (*P* < 0.05, Supplemental Fig. 3A).

### The tRNA^Met^ C4467A mutation helps induce mitochondrial membrane potential depolarization (ΔΨ_m_)

**Figure 4:**
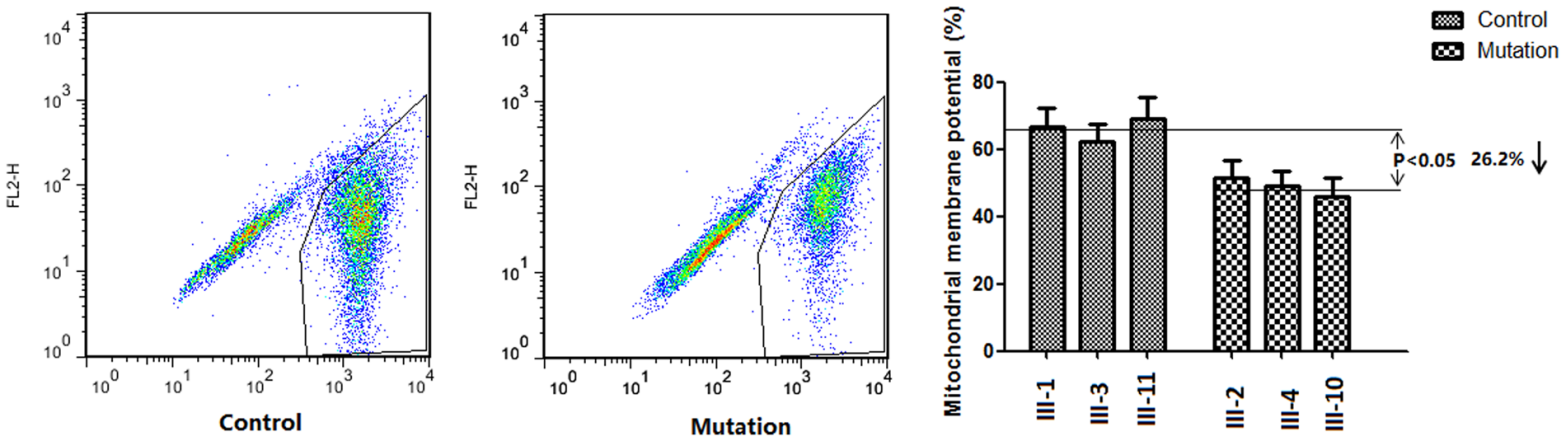
Mitochondrial membrane potential depolarization (ΔΨ_m_). The ΔΨ_m_ was measured in three mutant and three non-mutant cell lines, and a statistically significant decrease was observed. The results showed that the mean ΔΨ_m_ in mutant cell lines decreased 26.2% compared with the control cell lines (*P* < 0.05).

To further evaluate the effect of tRNA^Met^ C4467A on mitochondrial membrane potential depolarization, lymphocyte cells that had been pretreated with JC-10 were incubated for 10 min and then analyzed by flow cytometry. The mean mitochondrial membrane potential of the lymphocyte cell lines carrying the tRNA^Met^ C4467A mutation was decreased by 26.2% compared with the control group (Fig. 4; 48.67 ± 4.59 vs 65.98 ± 8.94,).

### The tRNA^Met^ C4467A mutation contributes to a decrease in mitochondrial oxygen consumption

**Figure 5:**
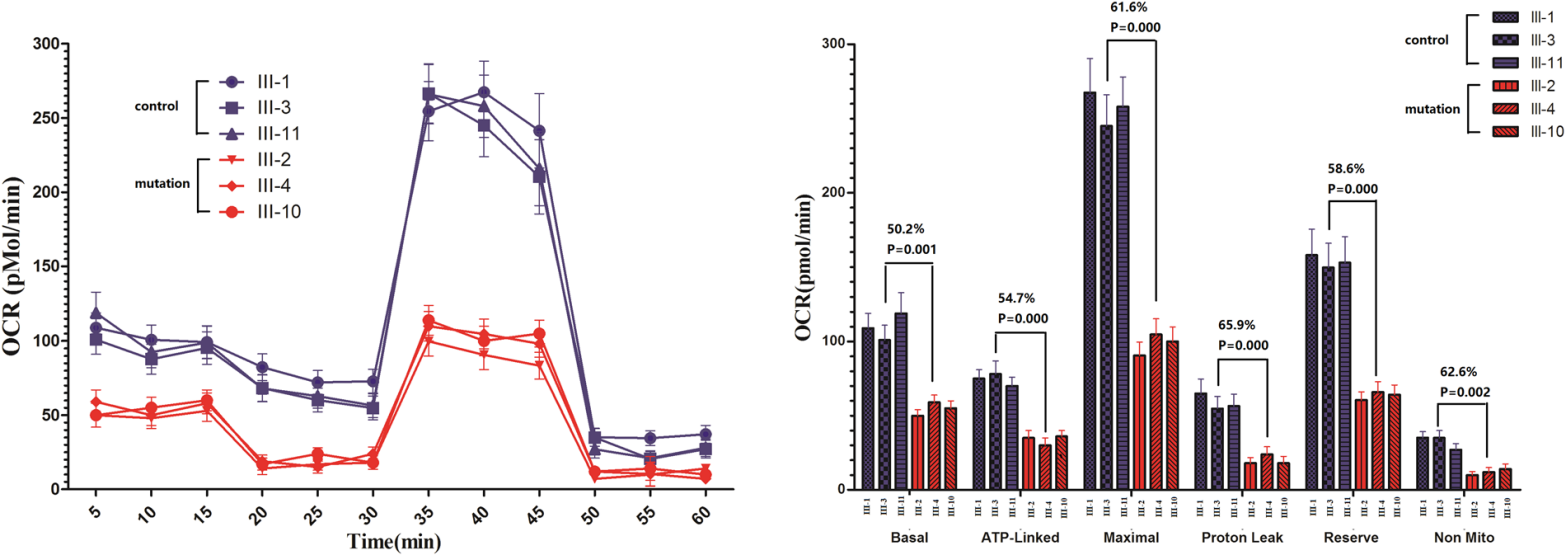
The tRNA^Met^ C4467A mutation contributes to a decrease of mitochondrial oxygen consumption. The basal OCR, ATP-linked OCR, proton leak OCR, maximal OCR, reserve capacity and non-mitochondrial decreases were 50.2%, 54.7, 61.6, 65.9, 58.6 and 62.6% compared with the control cell lines (P < 0.05), respectively.

Here, we evaluated the oxygen consumption rates (OCR) of lymphocyte cell lines that were derived from three tRNA^Met^ C4467A mutation-carrying individuals (III-2, 4, and 10) and three controls (III-1, 3, and 11) to evaluate whether the tRNA^Met^ C4467A mutation affects cellular bioenergetics. The basal OCR in mutant cell lines decreased 50.2% (*P* = 0.001, shown in Fig. 5) compared with that of control cell lines. To study whether the respiratory chain enzyme complexes were affected by the tRNA^Met^ C4467A mutation, OCR was evaluated after oligomycin (an ATP synthase inhibitor), FCCP (uncouples the mitochondrial inner membrane, allowing maximum electron flux through the electron transport chain), rotenone (a complex I inhibitor), or antimycin A (a complex III inhibitor). ATP-linked OCR, proton-leak OCR, maximal OCR, reserve capacity, and non-mitochondrial OCR that were associated with the basal OCR and the drug-insensitive OCR were compared between the mutant and non-mutant cell lines. These values decreased in mutant cell lines by 54.7%, 61.6%, 65.9%, 58.6%, and 62.6%, respectively, compared with the control cell lines (*P* < 0.05, Fig. 5).

### Caspase 3/7 activity evaluation

**Figure 6:**
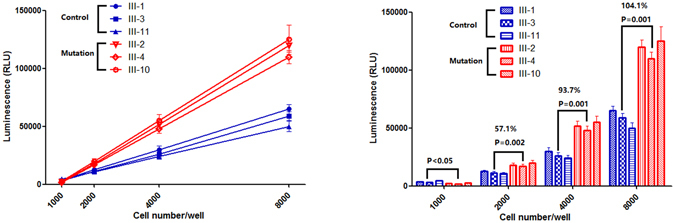
Caspase 3/7 activity evaluation. The lymphocyte cells lines were harvested and examined using a Caspase-Glo 3/7 assay kit according to the manufacturer’s instructions. Caspase-3/7 activity in the mutant cell lines was significantly increased by 57.1% in the 2 × 10^3^ cell group, 93.7% in the 4 × 10^3^ cell group, and 104.1% in the 8 × 10^3^ cell group compared with control cell lines (*P* < 0.05).

Cells were harvested, and caspase-3/7 activity was compared between the mutant and non-mutant cell lines. Activation of apoptosis was also represented by the activity of caspase-3/caspase-7. As shown in Fig. 6, the concentration gradient of 1 × 10^3^, 2 × 10^3^, 4 × 10^3^, and 8 × 10^3^ cells presented a linear relationship with caspase-3/caspase-7 activity both in tRNA^Met^ C4467A-mutated and control cell lines. Caspase-3/7 activity in the lymphocytes with the tRNA^Met^ C4467A mutation was significantly increased by 57.1% in the 2 × 10^3^ cell group, 93.7% in the 4 × 10^3^ cell group, and 104.1% in the 8 × 10^3^ cell group compared with the control cell lines (*P* < 0.05).

### Expression and colocalization of VDAC, Bax and AIF

**Figure 7:**
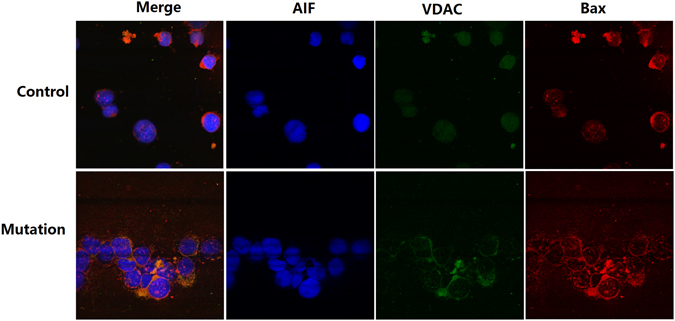
VDAC, BAX and AIF co-localization in lymphocytes by immunofluorescence. AIF is labeled with blue fluorescence, Bax is labeled with red fluorescence, and VDAC is labeled with green fluorescence. The control cell line exhibited no colocalization (yellow fluorescence) of Bax and VDAC or Bax and AIF (purple fluorescence). The mutant cell line showed the colocalization (yellow fluorescence) of Bax and VDAC but not the colocalization of Bax and AIF (purple fluorescence).

In control and mutated cell lines, confocal microscopy was used to observe the colocalization of voltage-dependent anion channel (VDAC, green fluorescence), Bax (red fluorescence) and apoptosis-inducing factor (AIF, blue fluorescence). There was no evidence of yellow fluorescence in the control group. In contrast, the mutated cells presented with yellow fluorescence (see Fig. 7) and showed the colocalization of Bax and VDAC. Both the control and mutated cell lines did not exhibit purple fluorescence, which indicated the lack of Bax (red fluorescence) and AIF (blue fluorescence) colocalization (Fig. 7).

**Figure 8:**
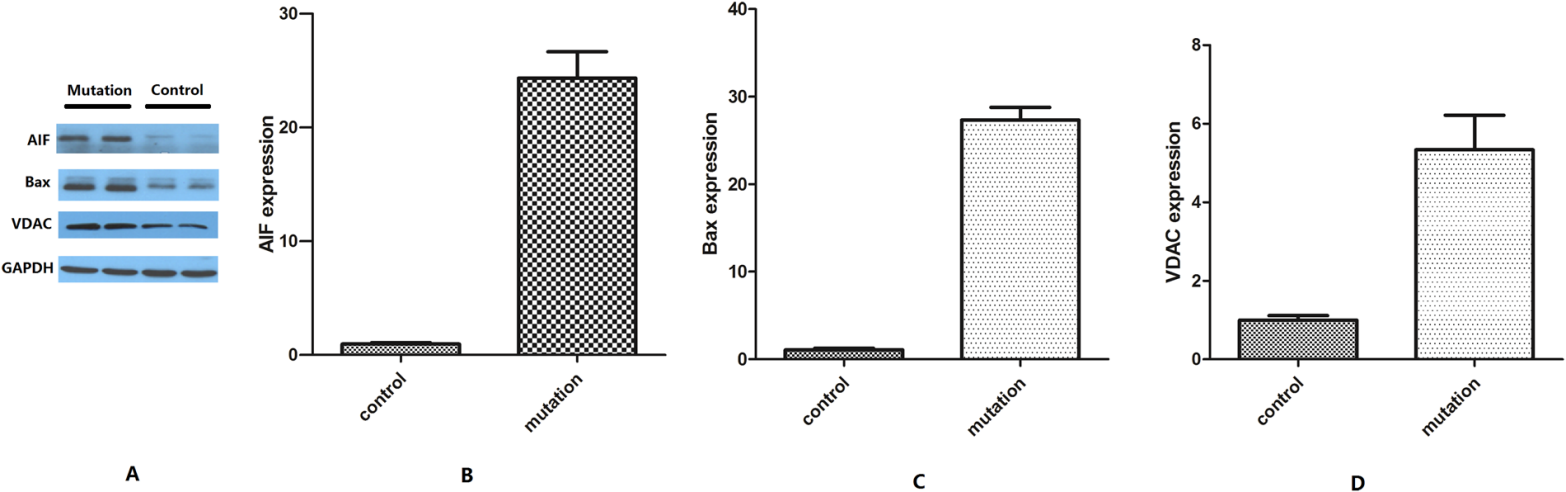
Western blotting of AIF, Bax and VDAC. The expression of AIF, Bax and VDAC increased in the mutant cell lines compared with the control cell lines (P < 0.05).

The protein expression of VDAC, Bax and AIF in the mutated cell line was higher than that in the control cell line (*P* < 0.05, Fig. 8). The increased expression of VDAC, Bax and AIF contributes to the increase of apoptosis.

## Discussion

Although many studies have revealed the genetics of mitochondrial disease, the pathophysiological mechanisms underlying essential hypertension remain unclear. Mitochondrial diseases are mitochondrial metabolic abnormalities caused by a variety of pathological changes, including changes in mitochondrial structure and function as well as mtDNA mutations, which are associated with disorders of the oxidative phosphorylation process and a reduction of overall cell function^16^. Several mitochondrial diseases with a maternal inheritance pattern have been reported, such as Leber hereditary optic neuropathy^17^ and noninsulin-dependent diabetes mellitus^18^. Additionally, many geriatric diseases are associated with pathological mutations of mtDNA, including Alzheimer’s disease^19^ and Parkinson’s disease^20^. Essential hypertension is influenced by multiple environmental and genetic factors, with the latter contributing to 20–50% of disease etiology.

In 2001, Watson *et al*.^21^ reported a double *ND3* A10398G Ddel *CO1* HaeIII T6620C/G6260A mutation in hypertensive black Americans with ESRD (end-stage renal disease). Another study by Wilson *et al*.^22^ documented a large family of metabolic defects caused by a mitochondrial tRNA^Ile^ 4291 T→C mutation. Additionally, we previously also identified excess maternal transmission of hypertension in several hypertensive families^11–14^. By far, the most widely studied mitochondrial diseases are those affecting single tRNA genes. Recently, Pro. Guan *et al*.^23^ studied the pathophysiology of the tRNA^Ala^ 5655 A→G mutation in hypertension. A failure in tRNA^Ala^ metabolism was shown to contribute to different reductions in six polypeptides encoded by mtDNA. The activity of the mitochondrial respiratory chain was decreased in the mutant cell lines, which was accompanied by decreases in ATP levels and mitochondrial membrane potential and an increase in the production of ROS. Therefore, the tRNA^Ala^ 5655A→G mutation may contribute to hypertension. However, more detailed effects of tRNA mutations on high BP are still unclear, partly because of ethical limitations on human material.

In this study, we are the first to report a novel tRNA^Met^ 4467C→A mutation in a Chinese family with maternally inherited hypertension. Biochemical testing showed a significant increase in blood glucose, TC, and LDL-c, and serum sodium as well as a decrease of potassium in maternal relatives compared with non-maternal relatives. Similarly, Wilson reported a large family with metabolic defects that were associated with the tRNA^Ile^ 4291 T→C mutation, in which maternal members presented with increased incidence of hypertension, hypercholesterolemia, and hypomagnesemia compared with non-maternal members. In this study, we also found that the average onset age tended to be earlier and that genetic factors contribute to the early onset age phenomenon.

In this study, the sequence analysis of the whole mitochondrial genome identified a novel mutation 4467C→A in tRNA^Met^ in a Chinese family. The results strongly suggest that this novel mutation may be involved in the pathogenesis of hypertension, as it is absent from 366 Chinese controls. The acceptor arm of the tRNA^Met^ is a 7-bp stem that is generated by the pairing of the 3′-terminal nucleotide with the 5′-terminal nucleotide. The 4467C→A mutation is in the second position from the 3′-terminal nucleotide. The CCA 3′-terminus is able to carry the amino acid. A subset of nucleotides is post-transcriptionally modified with a CCA triplet attached to the pre-tRNA 3′ end. In addition, the correctly folded and mature tRNAs are esterified by the corresponding aminoacyl-tRNA synthetase at the 3′ end with the cognate amino acid. Finally, protein synthesis is initiated after these tRNAs are transported to the ribosome by the translation elongation factor (EF)-Tu. The mutation may impair the cleavage of polycistronic mtRNA transcripts into mature mt-tRNA species at the tRNAase Z cleavage site. Thus, the point mutation located at the 3′ end is important for both tRNA synthesis and function^24^. Several studies have shown that pathogenesis-associated mutations in tRNAs could reduce the efficiency of precursor 3′-end cleavage using wild-type and pathogenesis-associated mutations in tRNA^Ser(UCN)^, tRNA^Ile^ and tRNA^Leu(UUR)^, which were at a similar position as tRNAMet4467C>A^25–28^. In the present study, phylogenetic analysis also demonstrated that the 4467C→A mutation site is highly evolutionarily conserved among species.

We established cell lines from three maternal and three non-maternal members of a Chinese family to study the effect of the 4467C→A mutation on mitochondrial function. ROS production in cells carrying the mutation was increased by 114.5% compared with that in control cell lines, while ATP production was decreased by 26.4%, and the mean mitochondrial membrane potential was decreased by 26.2%.

The mitochondrial electron transport system (ETS) was investigated by measuring OCR. As we know, the ATP-linked OCR and the reserve capacity reflect a “mitochondrial stress test,” which is evaluated based on the OCR in response to drugs, including oligomycin and FCCP. The basal OCR decreased by 50.2% in the mutant lymphocyte cell lines compared with controls, while ATP-linked OCR decreased by 54.7% in the mutant cell lines, which reflects the OCR of the coupling of mitochondrial oxidative phosphorylation. The maximal OCR, proton leak OCR, and reserve capacity OCR decreased by 61.6%, 65.9%, and 58.6%, respectively, in the mutant cell line, reflecting mitochondrial oxygen biogenesis dysfunction. Finally, significantly increased activation of caspase-3/7 was observed in the mutant cell lines, which is associated with the increased apoptosis of cells carrying the tRNA^Met^ C4467A mutation.

VDAC is the main channel by which ATP and other metabolites translocate between the mitochondria and the cytoplasm, and it plays an important role in the regulation of mitochondrial metabolism and cell growth; VDAC is located in the mitochondrial outer membrane^29^. Bax is a pro-apoptotic protein. Studies have shown that the binding of Bax to VDAC could lead to a change in mitochondrial membrane permeability, which significantly decreases the mitochondrial membrane potential, resulting in the release of apoptosis related factor (AIF) and cytochrome *c*, which promote apoptosis^30^. Our previous studies have also showed that the increased expression and colocalization of VDAC and Bax contribute to apoptosis^31^.

Due to the limitations of ethical human material and transmitochondrial animal models, we can only get peripheral blood lymphocytes from patients with mtDNA mutations, which is a challenge for all mtDNA-related diseases. Mitochondrial diseases presented with heteroplasmy, which indicates that both normal and mutant mtDNA coexist in one cell. In addition, the percentage of mutant mtDNA is different among different tissues, and different tissues show clinical symptoms at different thresholds. The percentage of mutant mtDNA in cardiomyocytes, neuronal cells and vascular endothelial cells are much higher than that in peripheral blood lymphocytes. However, the thresholds in these cells for the presentation of clinical symptoms are lower than that in lymphocytes. Therefore, if mtDNA mutations in lymphocytes could lead to increased ROS synthesis, decreased ATP synthesis and increased caspase3/7 apoptosis-associated activity, we could speculate that these changes would be more obvious in endothelial cells.

In conclusion, the present study identified a novel tRNA^Met^ C4467A mutation in a Han Chinese family with hypertension. Segregation analysis and pedigree mtDNA sequence analysis showed that this family presented with a maternally inherited mutation pattern. Functional analysis of lymphocyte cell lines carrying the tRNA^Met^ C4467A mutation showed that it contributes to mitochondrial dysfunction, including increased oxygen free radicals and cell apoptosis as well as decreased ATP synthesis and mitochondrial membrane potential, thereby causing mitochondrial oxygen consumption disorders. The expression of VDAC, Bax and AIF in mutant cell lines increased compared with that in control cell lines; additionally, there was an increase of VDAC and Bax colocalization, which contributes to cell apoptosis. Despite our findings that mitochondrial biogenesis dysfunction is associated with the tRNA^Met^ C4467A mutation, the exact mechanism remains unclear and should be examined in future studies.

## Methods

### Study design and patients

The protocols were approved by the ethics committee of the Chinese PLA General Hospital and performed in accordance with the approved JNC 8 guidelines and clinical regulations^32^. This Han Chinese family with maternally inherited hypertension was identified in the Cardiology Department of the Chinese PLA General Hospital. As part of a genetic screening program for hypertension, the protocol has been previously described in detail^11–14, 33^.

#### Measurements of blood pressure

One physician measured the systolic and diastolic blood pressures of all subjects using a mercury column sphygmomanometer. The first and the fifth Korotkoff sounds were indicative of systolic and diastolic blood pressure, respectively. The average of three measured blood pressure readings was taken as the examination blood pressure. Hypertension was defined according to the guidelines of the Joint National Committee on Detection, Evaluation and Treatment of High Blood Pressure (JNC VI) and the World Health Organization-International Society of Hypertension as a systolic blood pressure of 140 mmHg or higher and/or a diastolic blood pressure of 90 mmHg or greater or with a history of hypertension and under antihypertensive drug treatment.

A series of tests were conducted to exclude secondary hypertension. Bilateral renal and adrenal ultrasound and renal artery ultrasound were performed in all of the hypertensive patients in this study. The blood levels of renin, angiotensin, and aldosterone as well as the cortisol secretion rhythm were also evaluated.

In all cases, venous blood samples were drawn upon admission for biochemical examination. In addition, DNA samples of the maternal members from this family and 366 healthy controls were acquired for sequence analysis. Written informed consent was obtained from all participating subjects.

### mtDNA sequencing and sequence analysis

Genomic DNA was isolated using the Puregene DNA Isolation Kits according to the kit instructions (Gentra Systems, Minneapolis, MN). The entire mtDNA of all subjects, including hypertensive and control individuals, was PCR amplified in 24 overlapping fragments, with more detailed information described elsewhere^32, 34–36^.

### Lymphocyte cell lines and culture conditions

Lymphocyte cell lines were derived from three maternal members (III-2, 4, and 10) and three non-maternal members (III-1, 3, and 11) of this family (Fig. 1). Lymphoblastoid cell lines were first immortalized by transformation with the Epstein-Barr virus; more detailed methods were previously described^37^. Lymphoblastoid cell were cultured in RPMI 1640 medium (Gibco Company, USA) supplemented with 15% fetal bovine serum.

### Intracellular reactive oxygen species (ROS) production test

Intracellular ROS was evaluated by a 2′,7′-dichlorofluorescein-diacetate (DCFH-DA, 10 μM) liposoluble probe following the manufacturer’s instructions (Biyuntian, Beijing, China). The probe is hydrolyzed to DCFH, which is oxidized by ROS to fluorescent DCF. The fluorescence intensity is proportional to the level of ROS and was detected at 488 nm and 528 nm by flow cytometry (Becton Dickinson, USA).

### Intracellular ATP concentration

Samples of 1 × 10^3^, 2 × 10^3^, 4 × 10^3^, and 8 × 10^3^ lymphocytes from each group were homogenized in a protein extraction solution and centrifuged at 8,000 × *g* for 10 min; then, the supernatant was collected for ATP measurement using a bioluminescence-based cell viability assay (Promega Co.). Light emitted from the luciferase-mediated reaction was detected by a tube luminometer (BioTek, Germany).

### Mitochondrial membrane potential (ΔΨ_m_)

Lymphoblastoid cells were incubated with 5 μmol/L JC-10 (ABD Bioquest Inc., Sunnyvale, CA) for 10 min at 37 °C. Fluorescence intensities of JC-10 monomers and aggregates were measured by the FL1 (530/30 nm) and FL2 (585/42 nm) detectors of the flow cytometer, respectively. The JC-10 aggregate/monomer ratio is associated with the mitochondrial membrane potential intensity as previously described^38^.

### Mitochondrial oxygen consumption

The rates of oxygen consumption in lymphocyte cell lines were evaluated with a Seahorse Bioscience XF-96 extracellular flux analyzer (Seahorse Bioscience). The rates of O_2_ were determined under basal conditions and with various substrates and inhibitors, including oligomycin (1.5 μM), carbonyl cyanide p-(trifluoromethoxy) phenylhydrazone (FCCP) (0.5 μM), rotenone (1 μM), and antimycin A (1 μM), as detailed elsewhere^15, 39^.

### Caspase 3/7 activity evaluation

The level of apoptosis after treatment was assessed with the Caspase-Glo 3/7 assay kit (G8090, Promega, Madison, WI). A protein sample (1 mL) was diluted in 24 μL of assay buffer, and then, the manufacturer’s protocol was followed.

### VDAC, Bax and AIF colocalization and expression

Studies have shown that the binding of Bax to VDAC could lead to a change in mitochondrial membrane permeability, which promotes the release of AIF from the mitochondria to the nucleus. Therefore, we evaluated the expression of VDAC-1, Bax and AIF. Immunostaining was used to assess the co-localization of AIF, VDAC-1 and Bax polypeptides. Specimens were fixed (4% paraformaldehyde in PBS) and then blocked with 5% BSA for 15 min. Lymphocytes were incubated in blocking solution (10% BSA in PBS) for 1 h at 37 °C followed by polyclonal monoclonal anti-Bax (1:100), anti-VDAC (1:200), and monoclonal anti-AIF (1:200) primary antibody incubation overnight at 4 °C. The cells were washed and then incubated with rhodamine 123, FITC green and AMCA for 60 min at 37 °C, respectively. Then, slides were imaged using a confocal laser scanning system. Excitation-emission involved the use of an Argon 488 nm laser in conjunction with a 505–525-nm filter and a 350-nm filter^40^.

The cells were isolated for protein extraction, and the protein concentrations were determined as previously described^41^. Equivalent amounts of protein were separated by SDS-PAGE, transferred to a membrane, and then incubated with anti-VDAC (1:1000; Abcam) or anti-Bax (1:500, Abcam) or anti-AIF (1:1000, Abcam) primary antibodies, followed by goat anti-mouse, rabbit or goat IgG/HRP (1:1000; ZSGB Biotechnical Co.) secondary antibodies. Quantity One software (Bio-Rad, Hercules, CA, USA) was used to calculate the gray value and gray area of the protein bands. Each cell sample was measured three times, and the average value was recorded.

### Statistical analysis

The data are represented as the mean ± SEM. The independent samples *t*-test was used to compare the control and mutated cell lines. The statistical software GraphPad Prism (La Jolla, CA) was used for data analysis. All analyses were performed using SPSS 18.0 statistical software. *P* ≤ 0.05 was considered statistically significant.

## Electronic supplementary material

Supplementary Info File #1
