# Supplementary material for: Mitochondrial biogenesis dysfunction and metabolic dysfunction from a novel mitochondrial tRNAMet 4467 C>A mutation in a Han Chinese family with maternally inherited hypertension
Source: Sci Rep. 2017 Jun 8;7:3034. doi: 10.1038/s41598-017-03303-w (PMC5465199; doi:10.1038/s41598-017-03303-w)
Supplement: Supplementary file 1 — Supplementary Info File #1 [file 41598_2017_3303_MOESM1_ESM.pdf]

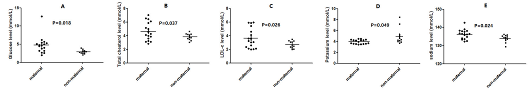

Supplement fig. S1 Biochemical examinations between maternal and non-maternal linearge of this Chinese Han family with hypertension. (A) Serum glucose level for individuals in maternal and non-maternal lineages of this family are shown and are significantly different (*P*=0.018). (B) Total cholesterol level for individuals in maternal and non-maternal lineages of this family are shown and are significantly different (*P*=0.037). (C) Low-density lipoprotein level for individuals in maternal and non-maternal lineages of this family are shown and are significantly different (*P*=0.026). (D) Serum sodium level for individuals in maternal and non-maternal lineages of this family are shown and are significantly different (*P*=0.049). (E) Serum potassium level for individuals in maternal and non-maternal lineages of this family are shown and are significantly different (P=0.024). ***(Title:Mitochondrial biogenesis and metabolic dysfunction with novel mitochondrial $tRNA^{Met}$ 4467C>A mutation in a Chinese Han family with maternal inheriting hypertension)***

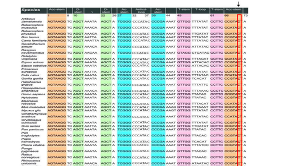

Supplement fig. S2 Alignment of tRNA^{Met} genes from different species. Square frame indicates the position of the Acc-stem 71 at the acceptor arm of tRNA^{Met}, correspond to the 4467 C>A. ***(Title:Mitochondrial biogenesis and metabolic dysfunction with novel mitochondrial tRNA^{Met} 4467C>A mutation in a Chinese Han family with maternal inheriting hypertension)***

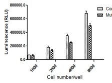

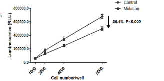

Supplement fig. S3 Measurement of ATP concentrations. The synthesis of ATP was compared with $1x10^3$, $2x10^3$, $4x10^3$ and $8x10^3$ cells in each group. The results showed that the synthesis of ATP was linearly related to the amount of cells. The synthesis of ATP was higher in control than mutation. (*P*<0.05) ***(Title:Mitochondrial biogenesis and metabolic dysfunction with novel mitochondrial tRNA^{Met} 4467C>A mutation in a Chinese Han family with maternal inheriting hypertension)***
